# Bone marrow dosimetric analysis of lymphopenia in patients with esophageal squamous cell carcinoma treated with chemoradiotherapy

**DOI:** 10.1002/cam4.4131

**Published:** 2021-08-07

**Authors:** Qian Wang, Qingtao Qiu, Zicheng Zhang, Jing Zhang, Guanghui Yang, Chengxin Liu, Baosheng Li

**Affiliations:** ^1^ Cheeloo College of Medicine Shandong University Jinan China; ^2^ Department of Radiation Oncology Shandong Cancer Hospital and Institute Shandong First Medical University and Shandong Academy of Medical Sciences Jinan China; ^3^ Department of Radiation Oncology Shenzhen Traditional Chinese Medicine Hospital The Fourth Clinical Medical of Guangzhou University of Chinese Medicine Shenzhen Guangdong China

**Keywords:** bone marrow, chemoradiotherapy, esophageal cancer, lymphocytes

## Abstract

**Background:**

We analyzed the relationship among peripheral blood lymphocytes, exposed sternum and vertebra body bone marrow (BM), and overall survival (OS) to find BM dosimetric parameters of lymphopenia during chemoradiotherapy (CRT) for patients with esophageal squamous cell carcinoma (ESCC).

**Methods:**

We examined 476 ESCC patients from January 2012 to January 2015, all of whom received concurrent or sequential CRT. Absolute lymphocyte counts (ALC) during radiotherapy (RT) of each patient were collected from the routine workup at the following RT times: pretreatment ALC (ALC0), at 1–5, 6–10, 11–15, 16–20, and 21–25, and more than 26 sessions (called ALC1–6, respectively). The sternum and vertebral body BM were delineated in accordance with uniform standards, and the irradiated volumes were calculated by dose‐volume histograms (DVH). The Kaplan–Meier method and Cox proportional hazards regression were used to analyze the survival of the patients. Comparisons of DVH were performed using the Mann–Whitney U test or two‐sample *t*‐test where appropriate.

**Results:**

A relative volume of sternum BM irradiated by more than 20 Gy could clearly affect the peripheral blood lymphocytes. The V20 of sternum BM and V50 of vertebra body BM were related to the OS of the patients, and the level of ALC2 (at 6–10 times of RT) could predict the outcomes of patients. The Cox regression analyses showed that the 218 patients with ALC2 ≥ 0.8 × 10^9^/L had a significantly higher OS (47.0 months vs. 30.9 months, *p* < 0.0001) than the 258 patients with ALC2 < 0.8×10^9^/L.

**Conclusion:**

In patients with ESCC, the relative volume of sternum BM irradiated by more than 20 Gy was associated with lymphocytes. Patients with ALC2 ≥ 0.8 × 10^9^/L had a significantly higher OS. The V20 of the sternum BM, the V50 of the vertebra body BM, and the level of ALC2 were significant prognostic factors in patients with ESCC.

## Graphical abstract

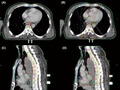

## 1 INTRODUCTION

Esophageal cancer (EC) is the eighth most common cancer worldwide and is the sixth most common cause of death from cancer.^1^ At present, neoadjuvant chemoradiotherapy (CRT) and radical concurrent CRT are the standard treatment of patients with advanced esophageal squamous cell carcinoma (ESCC) (National Comprehensive Cancer Network Guidelines Versions3.2020). Although these treatment methods have improved the overall survival (OS) rate relative to traditional treatments, they have also increased the occurrence of acute toxic effects, and the 5‐year survival rate is still less than 30%–40%.^2^

Clinical trials about immunotherapy, such as KEYNOTE‐181, ATTRACTION‐3, and KEYNOTE‐590, have shown it to be superior to chemotherapy in advanced EC whether in the first‐line or second‐line treatment.^3^, ^4^, ^5^ So research on the immune system is the key to further prolonging the survival rate of the patients. Lymphocytes as the primary mediators of cellular immunity in the human body are effective indicators in clinical work‐ups that reflect the immune function of patients with cancer. The occurrence, development, and prognosis of tumors are closely related to the immune function. When the immune function declines, tumor cells will escape the immune surveillance of the immune system and form solid tumors. However, as the most radiosensitive cells of the hematopoietic system, lymphocytes residing within or circulating through a radiation portal are frequently depleted by radiotherapy (RT), which indirectly interferes with the anti‐tumor process and affects the prognosis of the patients.^6^ Previous studies have shown that lymphopenia induced by radiation (LIR) can be used as a predictor of a poor outcome for many types of tumors, such as non‐small‐cell lung cancer, pancreatic cancer, breast cancer, glioma, and head and neck cancer.^7^, ^8^, ^9^, ^10^, ^11^

During the treatment of patients with EC, lymphocytes could be affected by a lot of factors. Current research has shown that the development of LIR is related to the irradiation of circulating blood and the dose of bone marrow within the RT target area.^12^, ^13^, ^14^, ^15^, ^16^ In MacLennan et al. study, their data strongly implicate dose received by circulating blood as a cause of lymphopenia.^12^ At the same time, a number of previous studies have also shown that the dose of bone marrow (BM) during RT is closely related to the occurrence of hematotoxicity in patients. BM as the main site of lymphocyte generation is very sensitive to radiation. In the process of treating EC patients with RT, the sternum and thoracic vertebra BM will inevitably be exposed to different doses of radiation as the target area changes. The hematopoiesis of the sternum and vertebral body BM accounts for 30% of the entire amount, so the generation of lymphocytes may be affected. In the study by Mell et al., cervical cancer patients with pelvic BM V10 ≥ 90% had higher rates of leukopenia and neutropenia than did patients with BM V10 < 90%.^15^ In addition, the mean dose and low‐dose radiation parameters (V5, V10, V15, V20) of whole bone or bone cavities of the lumbosacral spine were found to be correlated most significantly with HT3+ for squamous cell carcinoma of the anal canal.^16^ All of these data suggest a relationship between lymphocytes and dosimetric parameters of BM.

Based on the previous findings described above, this study aimed to explore the relationships between the sternum and vertebra body BM to the lymphocytes and OS of EC patients to analyze dosimetric predictors of lymphopenia during CRT for patients with ESCC.

## 2 METHODS

### 2.1 Patients and clinical data

This is a retrospective study. The ethics committee of Shandong Cancer Hospital and Institute approved this study. The study was conducted in agreement with the Declaration of Helsinki. And all the patients signed a consent to share their clinical data and information for clinical studies. Patients with histologically confirmed ESCC who received CRT at Shandong Cancer Hospital and Institute Affiliated with Shandong First Medical University between January 2012 and January 2015 were considered to be eligible. Considering the EC, staging criteria have slight differences in recent years, we reclassified the disease stage of the patients according to the eighth edition of the American Joint Committee on Cancer's (AJCC) Cancer Staging Manual.^17^ Inclusion criteria: (1) Karnofsky Performance Status (KPS) ≥70, pathologically confirmed patients with ESCC who received CRT; (2) no other tumors, no previous history of chemotherapy or RT; (3) survival period greater than 6 months; (4) normal blood cell level before treatment, no other immune diseases, no immune‐related drugs (suppression or enhancement). Exclusion criteria: (1) patients with incomplete case data who could not be reclassified as to disease stage; (2) the total dose of RT did not reach the established radical dose; (3) a lack of blood routine results during RT ≥2 times; (4) poor physical condition with severe underlying disease; (5) chronic inflammatory or autoimmune disease; (6) blood transfusion within the last 3 months.

We collected the following information from all of the patients: age, sex, KPS, tumor location, tumor length, differentiation, and Primary Tumor, Regional Lymph Nodes, Distant Metastasis (TNM) stage. The absolute value of lymphocytes (ALC) was collected within 1 week of starting RT, and then at 1–5, 6–10, 11–15, 16–20, 21–25, and more than 26 sessions of RT (referred to as ALC0, ALC1–6, respectively). One missing value was allowed during the whole treatment. Meanwhile, we also obtained the maximum, minimum, rate of decline, and the average value of lymphocytes. After the entire treatment was completed, the patients were followed‐up every 2 months for half a year. If their condition was stable, the follow‐up time was prolonged to every 3–6 months, and the overall follow‐up was for at least 5 years. The study endpoints were overall survival (OS) (time from the end of the treatment to death from any cause or the last follow‐up time).

### 2.2 Radiotherapy and chemotherapy

The target of RT was delineated on the eclipse system as follows. Gross Tumor Volume (GTV): Combined with CT/PET‐CT or titanium clip markers under the endoscope to identify the primary tumors and positive lymph node areas; Clinical Target Volume (CTV): Add the high‐risk areas of lymphatic drainage on the basis of GTV; Planning Target Volume (PTV): 0.8 cm was added in the horizontal directions and 3 cm expansion in the axial direction to account for setup uncertainty and organ motion. Radiation dose: 50–60 Gy (delivered in 25–33 fractions of 1.8–2.0 Gy/fraction). Normally endangered organs: total lung V20 ≤ 35%, V5 ≤ 65%, Dmean ≤20 Gy; heart Dmean ≤35 Gy; spinal cord Dmax ≤45 Gy. In addition, all patients received platinum‐based chemotherapy with paclitaxel or fluorouracil every 21–28 days. The chemotherapy drugs were given according to the standard dose. Concurrent CRT means chemotherapy drugs at intervals of 21–28 days were given at the beginning of RT. Nonconcurrent CRT was mainly sequential and induction CRT.

### 2.3 Delineation of sternum and vertebra body BM

**FIGURE 1:**
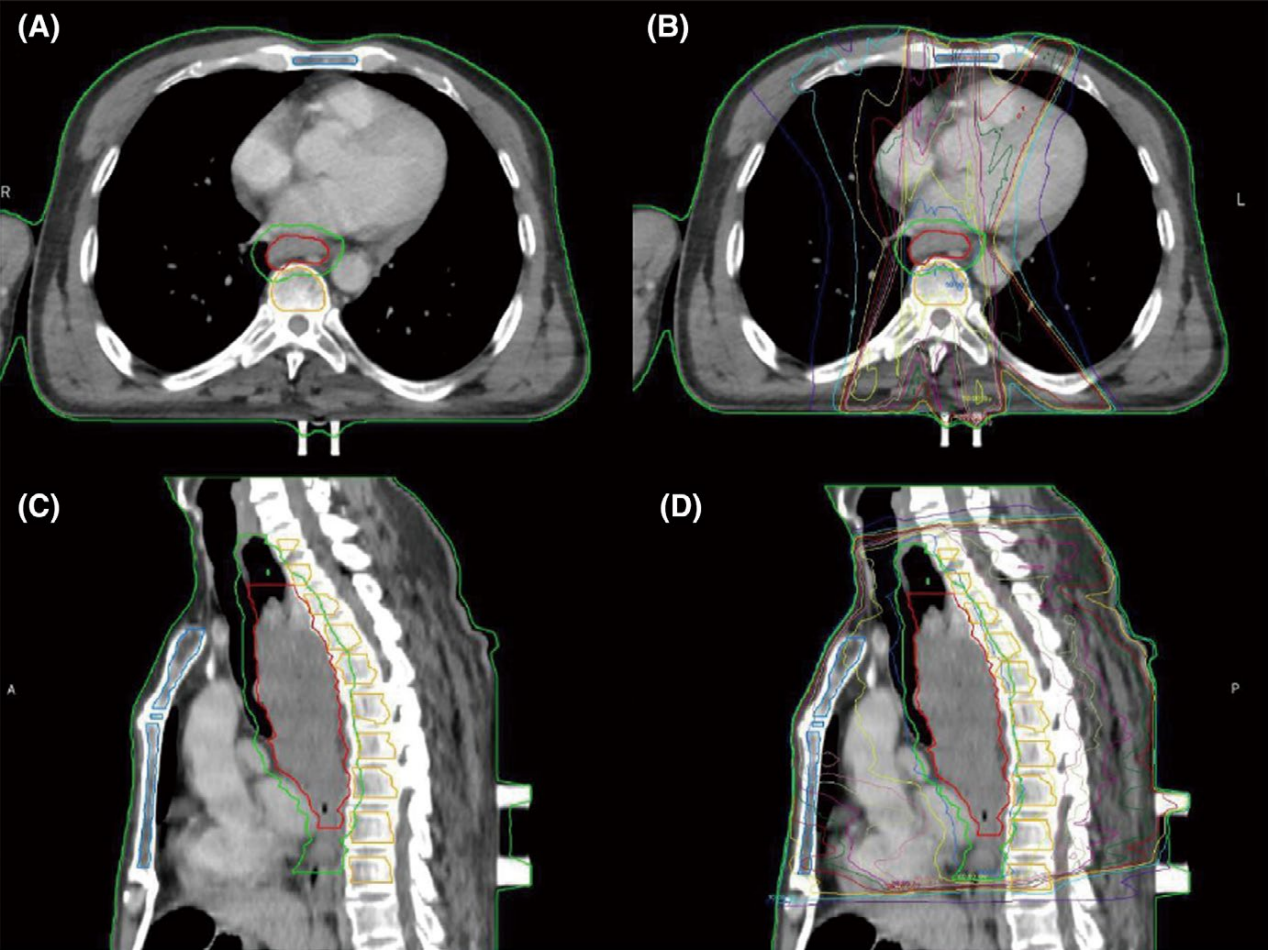
Delineation on cross‐sectional (A, B) and sagittal planes (C, D) CT. (B) and (D) show the isodose line of target. Structures included tumor PTV (green), GTV (red), vertebra body BM (yellow), and sternum BM (blue)

We transferred the CT images of all of the patients to the MIM (6.8.2) system and the delineation process was completed on the mediastinal window. Considering that the hematopoietic site of adults is mainly in the BM cavity, the boundary of bone and marrow is distinguished by the sudden change of CT value at the critical area. Combined bone windows (W2000Hu, L500Hu) with mediastinal window (W250Hu, L50Hu), we delineate the BM according to the following criteria as shown in (Figure 1). Sternum BM: (1) upper boundary is the sternum notch, lower boundary is the xiphoid process, the circumference is the junction with the bone; (2) due to the sternum angle being indistinguishable from the surrounding costal cartilage, the delineation of this area was done under the guidance of two imaging physicians. Vertebral body BM: (1) upper and lower bounds are the projection range of the 10% isodose line of PTV on the vertebrae, and the circumference is the junction with the bone; (2) avoid delineation of intervertebral discs and pyramidal attachments based on the continuity and anatomical structure of the spine.

### 2.4 Statistical analysis

The characteristics of the patients were described by descriptive statistics. Survival was estimated by the Kaplan–Meier method and survival curve comparisons were performed using the log‐rank test. Multiple logistic regression analysis was performed to correlate the clinical characteristics and OS. Propensity score matching was used to compensate for the differences in baseline characteristics. The cut‐off value of the lymphocytes was obtained by X‐tile software. Chi‐square tests were used to analyze the differences between group 1 and group 2. Comparisons of DVH were performed using the Mann–Whitney U test or two‐sample *t*‐test, where appropriate. All statistical analyses were performed using Statistical Package for the Social Sciences, SPSS software 22.0, and *p* < 0.05 was considered to be significant.

## 3 RESULTS

### 3.1 Basic characteristics

After 5 years of follow‐up, 476 patients were recruited into this study, including 367 men and 109 women. As shown in Table 1, the median age of all patients was 63 and 64.5% were over 60 years. We put stage I and stage II patients into one group for analysis. Stage I and II, III, and IV were administered to 11.3%, 61.6%, and 27.1% of the patients, respectively. Stage I and II patients were given CRT treatment due to the following reasons: contraindicated for surgery due to a poor physical condition (11 patients), cervical EC (16 patients), and refusal of surgery (27 patients). Only 12.2% of patients were treated with concurrent CRT, while 87.8% had sequential CRT. The average courses of chemotherapy were 4.2 courses, and the average dose of RT was 59.3 Gy. Most patients (65.8%) were treated with intensity‐modulated radiation therapy (IMRT). The median OS of the patients was 28.4 months, and the 1‐year, 3‐year, and 5‐year OS rates were 82.1%, 32.8%, and 3.8%, respectively.

**TABLE 1:** Relationship between basic clinicopathological characteristics of patients and OS

Characteristics	Number (%) (Total patients = 476)	*p* value
Age (median [range])	63 (37–85)	
Age(<60 vs. ≥60)	169 (35.5) versus 307 (64.5)	0.186
Gender (male vs. female)	367 (77.1) versus 109 (22.9)	0.141
KPS (70 vs. 80 vs. 90)	15 (3.2) versus 235 (49.4) versus 226 (47.5)	0.011*
Tumor location (upper vs. middle vs. lower)	196 (41.2) versus 205 (43.1) versus 75 (15.8)	0.245
TNM (I, II vs. III vs. IV)	54 (11.3) versus 293 (61.6) versus 129 (27.1)	<0.0001*
RT‐technology (IMRT vs. CRT)	313 (65.8) versus 163 (34.2)	0.232
Dose(50 group vs. 60 group)	74 (15.5) versus 402 (84.5)	0.828
RT‐pattern (Concurrent vs. Non‐concurrent)	58 (12.2) versus 418 (87.8)	0.173
Chemotherapy (Paclitaxel group vs. 5‐FU group)	276 versus 200	0.734
Chemotherapy cycle(1–2 vs. 3–4 vs. 5–6 vs. >6)	104 (24.2) versus 183 (38.9) versus 143 (37.0) versus 46 (9.7)	0.154
ALC0		0.636
ALC1		0.261
ALC2		0.025*
ALC3		0.961
ALC4		0.325
ALC5		0.112
ALC6		0.978
CEA before treatment		0.204
CYFRA before treatment		0.002*

Abbreviations: ALC, absolute value of lymphocytes; CRT, chemoradiotherapy; IMRT, Intensity‐modulated radiation therapy; KPS, Karnofsky Performance Status; RT, radiotherapy; TNM, Primary Tumor, Regional Lymph Nodes, Distant Metastasis.

* Statistically significant.

### 3.2 Correlation between basic clinicopathological characteristics and OS

The correlations between the basic clinicopathological characteristics and OS were analyzed by multiple logistic regression, and KPS (*p* = 0.011), TNM staging (*p* < 0.0001), ALC2 (6–10 sessions of RT) (*p* = 0.025), and the pretreatment CYFRA level (*p* = 0.002) had a statistical correlation with OS (Table 1). Moreover, there was no significant correlation between OS and chemotherapy, minimum value of lymphocytes, the maximum value of ALC, the drop rate, ALC0, 1, 3–6, sex, age, RT dose, or RT technique (*p* > 0.05). We obtained the ALC2 cut‐off value (0.8 × 10^9^/L) using X‐tile. All patients were divided into two groups: less than 0.8 × 10^9^/L group (group 1) and the greater than or equal to 0.8 × 10^9^/L group (group 2). The patients in group 1 were matched 1:1 to the patients in group 2 according to their propensity score using the global optimum method, 55 pairs of patients were matched finally.

### 3.3 Comparison of the differences between the two groups

**FIGURE 2:**
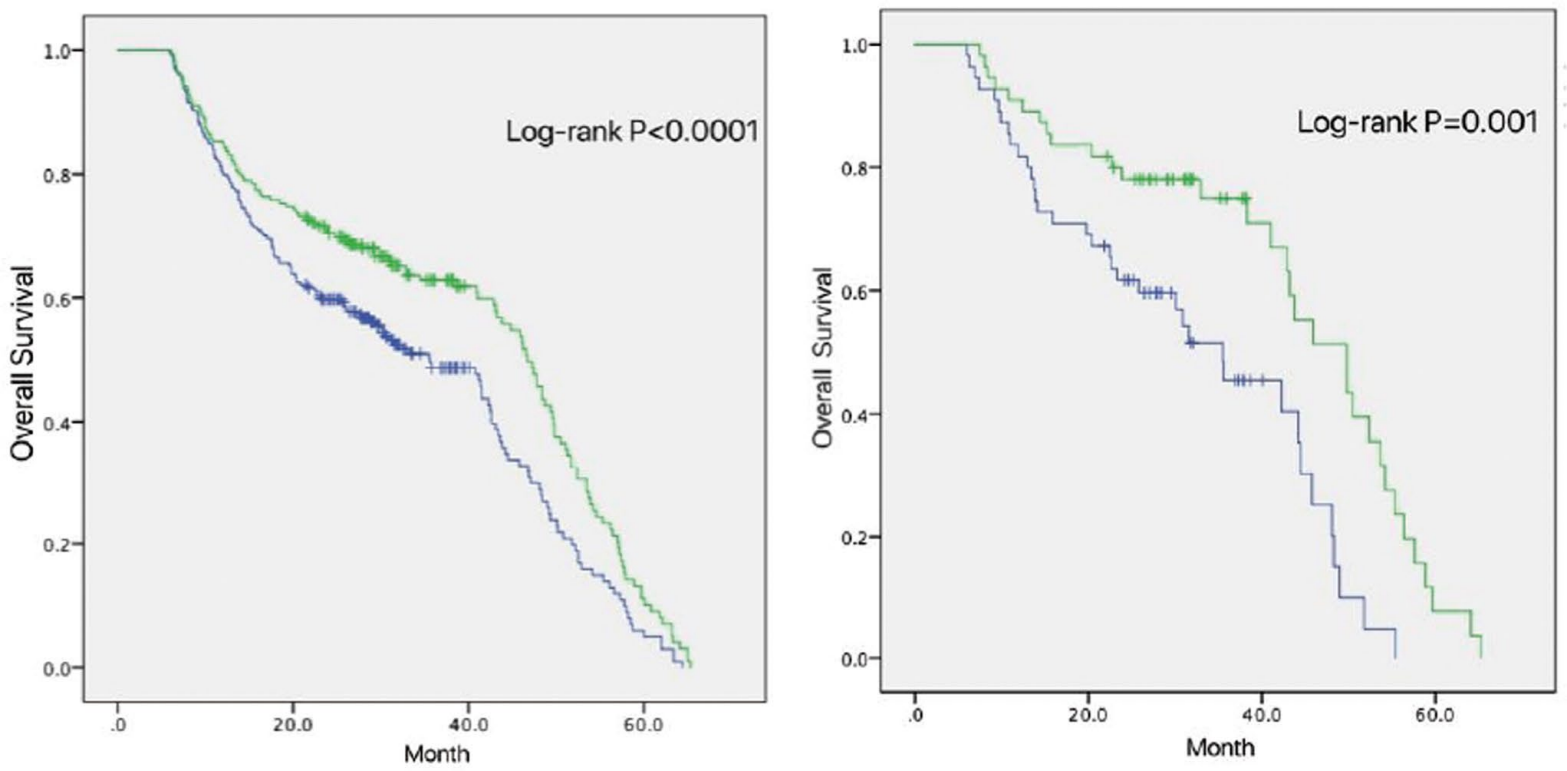
Kaplan–Meier curves showing overall survival between group1 (blue line) and group2 (green line) before (left) and after (right) Propensity score matching

**FIGURE 3:**
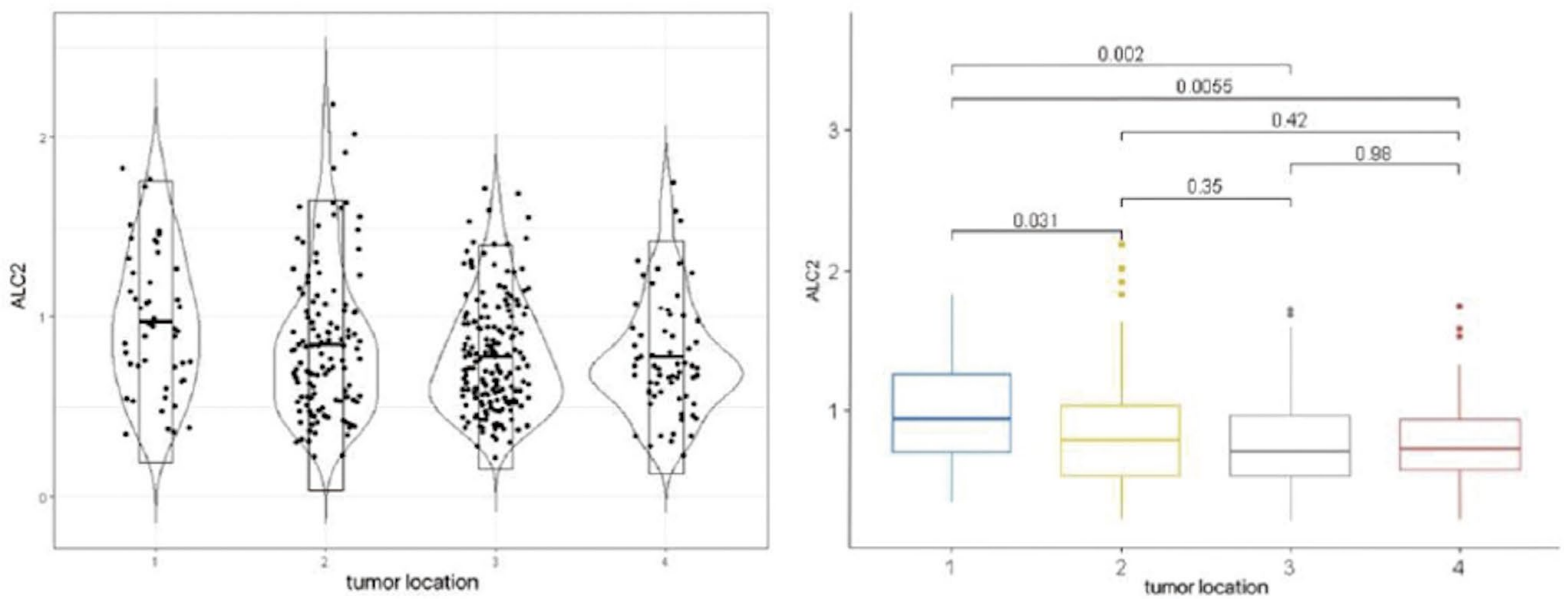
The left shows data distribution of ALC 2 in patients with tumor location in each group. The right shows the differences of ALC 2 in patients with tumor location in each group by pairwise comparison. (1‐Cervical EC; 2‐Upper thoracic EC; 3‐Middle thoracic EC; 4‐Lower thoracic EC)

Group 1 showed a significantly inferior median OS compared with group 2 (30.9 months vs. 47.0 months, *p* < 0.0001) (Figure 2). After matching for the other confounding factors, the median OS of the two groups were 35.5 versus 49.8 months (*p* = 0.001). In Table 2, we can see there were significant differences between the two groups for tumor location, RT pattern, chemotherapy, and CYFRA level before treatment (*p* < 0.05). On further analysis of the tumor location, we found that middle and lower thoracic EC were more likely to be associated with a decrease in peripheral blood lymphocytes than cervical EC (Figure 3).

**TABLE 2:** Comparative analysis of patient clinicopathological characteristics from unmatched and propensity‐matched groups

Characteristic	Before matching			After matching		
	LC < 0.8 (*n *= 258) (%)	LC ≥ 0.8 (*n* = 218) (%)	*p* value	LC < 0.8 (*n* = 55) (%)	LC ≥ 0.8 (*n* = 55) (%)	*p* value
Age (average)	61.8	62.5	0.0971	63.8	63.0	1
Age <60	97 (20.4)	72 (15.1)		16 (14.5)	16 (14.5)	
Age ≥60	161 (33.8)	146 (30.7)		39 (35.5)	39 (35.5)	
Gender			0.500			0.654
Male	202 (42.4)	165 (34.7)		41 (37.3)	43 (39.1)	
Female	56 (11.8)	53 (11.1)		14 (12.7)	12 (10.9)	
KPS			0.124			0.615
70	10 (2.1)	5 (1.1)		2 (1.8)	3 (2.7)	
80	136 (28.6)	99 (20.8)		31 (28.2)	26 (23.6)	
90	112 (23.5)	114 (23.9)		22 (20.0)	26 (23.6)	
Tumor location location			<0.0001*			0.496
Upper (cervical and upper thoracic)	92 (19.3)	104 (21.8)		21 (19.1)	20 (18.2)	
Middle thoracic	119 (25.0)	86 (18.1)		23 (20.9)	28 (25.5)	
Lower thoracic	47 (9.9)	28 (5.9)		11 (10.0)	7 (6.4)	
Tumor lengthLength (cm)			0.634			0.603
1–5	167 (35.1)	138 (29.0)		37 (33.6)	38 (34.5)	
5.1–10	84 (17.6)	77 (16.2)		17 (15.5)	17 (15.5)	
10.1–15	6 (1.3)	2 (0.4)		1 (0.9)	0	
15.1–20	1 (0.2)	1 (0.2)		0	0	
TNM			0.224			0.699
I, II	29 (6.1)	25 (5.3)		6 (5.5)	7 (6.4)	
III	149 (31.3)	144 (30.3)		40 (36.4)	36 (32.7)	
IV	80 (16.8)	49 (10.3)		9 (8.2)	12 (10.9)	
cT stage			0.105			0.823
T1, T2	29 (6.1)	22 (4.6)		5 (4.5)	6 (5.5)	
T3	175 (36.8)	150 (31.5)		40 (36.4)	37 (33.6)	
T4	54 (11.3)	46 (9.7)		10 (9.1)	12 (10.9)	
cN stage			0.951			0.792
N0	35 (7.4)	30 (6.3)		9 (8.2)	8 (7.3)	
N+	223 (46.8)	188 (39.5)		46 (41.8)	47 (42.7)	
RT‐technology			0.946			1
IMRT	170 (35.7)	143 (30.0)		34 (30.9)	34 (30.9)	
CRT	88 (18.5)	75 (15.8)		21 (19.1)	21 (19.1)	
Dose (Gy)			0.463			0.376
50 group	43 (9.0)	31 (6.5)		8 (7.3)	5 (4.5)	
60 group	215 (45.2)	187 (39.3)		47 (42.7)	50 (45.5)	
RT‐pattern			0.036*			0.808
Concurrent	24 (5.0)	34 (7.1)		45 (40.9)	44 (40.0)	
Non‐concurrent	234 (49.2)	184 (38.7)		10 (9.1)	11 (10.0)	
Chemotherapy			0.024*			0.251
Paclitaxel+	166 (34.9)	118 (24.8)		22 (20.0)	28 (25.5)	
5‐FU+	92 (19.3)	100 (21.0)		33 (30.0)	27 (24.5)	
Chemotherapy cycle			0.658			0.763
1–2	55 (11.6)	49 (10.3)		16 (14.5)	13 (11.8)	
3–4	94 (19.7)	89 (18.7)		21 (19.1)	21 (19.1)	
5–6	82 (17.2)	61 (12.8)		18 (16.4)	21 (19.1)	
>6	27 (5.7)	19 (4.0)		0	0	
rhG‐CSF			0.602			0.848
Use rhG‐CSF	193 (74.8)	158 (72.5)		32 (58.2)	30 (54.5)	
Non‐use rhG‐CSF	65 (25.2)	60 (27.5)		23 (41.8)	25 (45.5)	
CEA			0.674			0.644
<1.6	61 (12.8)	48 (10.1)		13 (11.8)	11 (10.0)	
≥1.6	197 (41.4)	170 (35.7)		42 (38.2)	44 (40.0)	
CYFRA			0.018*			0.297
<6.4	223 (46.8)	203 (42.6)		49 (44.6)	52 (47.3)	
≥6.4	35 (7.4)	15 (3.2)		6 (5.4)	3 (2.7)	
Differentiation			0.317			0.915
hyper	18 (3,8)	23 (4.8)		4 (3.6)	5 (4.5)	
Middle	25 (5.3)	24 (5.0)		8 (7.2)	7 (6.3)	
lower	215 (45.2)	171 (35.9)		43 (39.1)	43 (39.1)	

Abbreviations: ALC, absolute value of lymphocytes; CRT, chemoradiotherapy; IMRT, Intensity‐modulated radiation therapy; KPS, Karnofsky Performance Status; LC, lymphocytes; RT, radiotherapy; TNM, Primary Tumor, Regional Lymph Nodes, Distant Metastasis.

* Statistically significant.

### 3.4 Correlation between the sternum and vertebral body BM and OS

The dose‐relative/absolute volume histograms of all patients could be obtained after drawing the sternum and vertebral body BM according to the criteria mentioned above. The results shown in Table 3 were obtained by Cox proportional hazard model: the relative and absolute V20 of the sternum BM were both statistically correlated with OS (*p* = 0.044; *p* = 0.027), and the relative and absolute V50 of the vertebra body BM were also statistically correlated with OS (*p* = 0.027; *p* = 0.024).

**TABLE 3:** Relationship between dose‐volume of BM and OS

Parameters	HR (CI 95%)	*p* value	Parameters
RV5 Gy	sternum	1.05 (0.90–1.21)	0.515
	vertebra body	0.92 (0.68–1.25)	0.594
RV10 Gy	sternum	0.96 (0.85–1.09)	0.581
	vertebra body	1.10 (0.87–1.40)	0.414
RV15 Gy	sternum	1.12 (0.99–1.26)	0.051
	vertebra body	1.02 (0.81–1.29)	0.878
RV20 Gy	sternum	0.92 (0.86–0.99)	0.044*
	vertebra body	0.99 (0.79–1.25)	0.950
RV25 Gy	sternum	1.03 (0.97–1.09)	0.414
	vertebra body	0.97 (0.83–1.13)	0.680
RV30 Gy	sternum	1.01 (0.96–1.06)	0.652
	vertebra body	1.05 (0.92–1.20)	0.482
RV35 Gy	sternum	0.97 (0.93–1.01)	0.122
	vertebra body	0.99 (0.92–1.06)	0.731
RV40 Gy	sternum	1.03 (0.99–1.07)	0.178
	vertebra body	0.99 (0.94–1.04)	0.675
RV45 Gy	sternum	0.99 (0.95–1.02)	0.463
	vertebra body	0.97 (0.91–1.02)	0.233
RV50 Gy	Sternum	1.01 (0.99–1.04)	0.428
	vertebra body	1.04 (1.00–1.08)	0.027*
AV5 Gy	sternum	1.00 (0.78–1.29)	0.991
	vertebra body	0.92 (0.70–1.21)	0.554
AV10 Gy	sternum	0.84 (0.47–1.53)	0.575
	vertebra body	1.09 (0.70–1.69)	0.716
AV15 Gy	sternum	1.66 (0.90–3.04)	0.102
	vertebra body	0.99 (0.66–1.49)	0.966
AV20 Gy	sternum	0.59 (0.37–1.94)	0.027*
	vertebra body	1.06 (0.72–1.54)	0.781
AV25 Gy	sternum	1.34 (0.95–1.91)	0.100
	vertebra body	0.91 (0.72–1.15)	0.430
AV30 Gy	sternum	0.97 (0.77–1.24)	0.823
	vertebra body	1.10 (0.91–1.33)	0.329
AV35 Gy	sternum	0.86 (0.72–1.03)	0.102
	vertebra body	0.99 (0.90–1.10)	0.826
AV40 Gy	sternum	1.18 (0.99–1.38)	0.053
	vertebra body	0.97 (0.90–1.04)	0.381
AV45 Gy	sternum	0.90 (0.76–1.06)	0.192
	vertebra body	0.96 (0.89–1.04)	0.284
AV50 Gy	sternum	1.04 (0.94–1.14)	0.440
	vertebra body	1.06 (1.01–1.11)	0.024*

Abbreviations: AVxGy, absolute volume irradiated by xGy; BM, bone marrow; HR, hazard ratio; OS, overall survival; RVxGy, relative volume irradiated by xGy.

* Statistically significant.

### 3.5 Relationship between the sternum and vertebra body BM and lymphocytes

**FIGURE 4:**
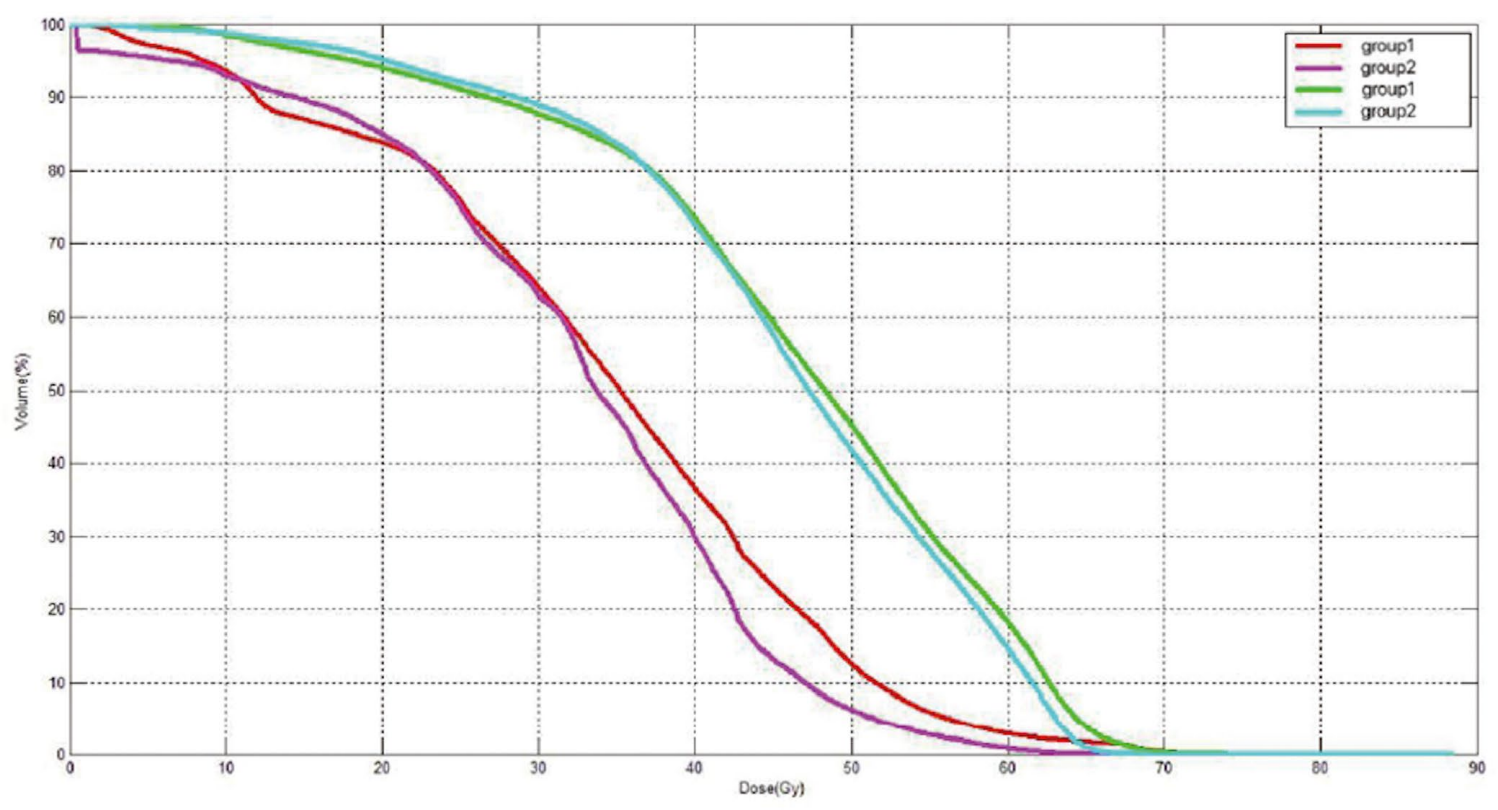
DVH of sternum BM (two red lines) and vertebra body BM (two green lines) of two groups after Propensity score matching, four solid lines is the mean values of each group

To analyze the relationship between the sternum and vertebra body BM and lymphocytes, we compared the differences of exposed BM between the two groups of patients divided according to the cutoff value of the lymphocytes. After propensity score matching (Figure 4), it was concluded that there was a significant difference in the relative volume of the sternum BM irradiated by more than 20 Gy between the two groups (*p* < 0.05), but there was no significant difference for vertebral body BM (*p* > 0.05; Table 4).

**TABLE 4:** Relative volume of the sternum, vertebra body BM between the two groups

Parameters		Relative volume	*p* value
RV5 Gy	S1 versus S2	96.5 versus 95.0	0.221
	V1 versus V2	99.9 versus 99.6	0.488
RV10 Gy	S1 versus S2	94.0 versus 93.7	0.134
	V1 versus V2	99.0 versus 99.2	0.859
RV15 Gy	S1 versus S2	86.5 versus 90.0	0.094
	V1 versus V2	95.6 versus 97.5	0.699
RV20 Gy	S1 versus S2	83.5 versus 85.0	0.033*
	V1 versus V2	94.0 versus 95.5	0.734
RV25 Gy	S1 versus S2	75.0 versus 75.0	0.002*
	V1 versus V2	91.0 versus 92.0	0.802
RV30 Gy	S1 versus S2	64.0 versus 63.0	0.0004*
	V1 versus V2	87.5 versus 89.1	0.782
RV35 Gy	S1 versus S2	50.8 versus 46.4	<0.0001*
	V1 versus V2	83.5 versus 83.5	0.922
RV40 Gy	S1 versus S2	30.0 versus 36.6	<0.0001*
	V1 versus V2	73.5 versus 73.3	0.888
RV45 Gy	S1 versus S2	23.5 versus 13.5	<0.0001*
	V1 versus V2	60.0 versus 57.5	0.789
RV50 Gy	S1 versus S2	6.0 versus 12.5	<0.0001*
	V1 versus V2	45.0 versus 42.0	0.584
RV55 Gy	S1 versus S2	2.5 versus 5.5	<0.0001*
	V1 versus V2	30.5 versus 37.5	0.669
RV60 Gy	S1 versus S2	1.0 versus 3.0	<0.0001*
	V1 versus V2	18.0 versus 15.0	0.351

Abbreviations: BM, bone marrow; RVxGy, relative volume irradiated by xGy; S1, sternum of group1; S2, sternum of group2; V1, vertebra body of group1; V2, vertebra body of group2.

* Statistically significant.

## 4 DISCUSSION

The results of this study indicated that the relative volume of sternum BM irradiated by more than 20 Gy could affect the peripheral blood lymphocytes. Patients with ALC2 ≥ 0.8 × 10^9^/L had a significantly higher OS. The V20 of the sternum BM, the V50 of the vertebra body BM, and the level of ALC2 were significant prognostic factors in ESCC patients. Based on these findings, we suggest that in clinical work, while ensuring the target treatment dose of patients with ESCC, the V20 of the sternum BM and the V50 of the vertebra body BM should be reduced as much as possible, it may be ultimately beneficial to the survival of the patients.

During this study, 350 patients suffered leukopenia during treatment, clinically treated with Recombinant Human Granulocyte Colony Stimulating Factor (rhG‐CSF) injection. In order to observe whether it affects the results, we made the corresponding analysis. In Table 2, we can see that there is no significant statistical difference between the two groups of patients on whether to use rhG‐CSF, that is, the use of rhG‐CSF has little impact on the grouping of patients. What is more, in patients with ESCC treated with CRT, we found that ALC2 can predict the patients’ outcomes. Some previous studies have also reported lymphocytes are correlated with the prognosis of patients. Deng et al. demonstrated that significant G4 lymphopenia (<200 cells/ml) during CRT was an independent predictor of survival outcomes of patients with EC.^18^ The minimum ALC may be a prognostic factor indicating a worse outcome for nasopharyngeal cancer and hepatocellular carcinoma.^19^, ^20^ Pretreatment lymphopenia is a predictor of a good outcome as well as a predictive factor for tumor response and chemotherapy‐related hematological toxicity in metastatic ESCC.^21^ Furthermore, we also found that middle and lower thoracic EC were more likely to cause a decrease in peripheral blood lymphocytes than cervical EC. This conclusion is consistent with the previous study,^18^ and it may be that the lung, heart, and spleen are more affected by radiation during RT of middle and lower thoracic EC than cervical EC.^22^, ^23^, ^24^ As the distal esophageal location passes across the heart, a significant pool of lymphocytes will be exposed to radiation treatment for distal esophageal tumors.^23^ Liu et al. showed that higher spleen irradiation doses were significantly correlated with a lower Min ALC during RT for hepatocellular carcinoma.^24^ Here, based on the current evidence, the effects of the distribution of heart and BM irradiation on lymphocytes are difficult to distinguish. And our another prospective study of delineating heart on Magnetic Resonance Imaging (MRI) is under way, which may bring a reasonable explanation.

We found that the relative volume of sternum BM irradiated by more than 20 Gy could obviously affect the peripheral blood lymphocytes in patients with ESCC. Miyoshi N et al. have shown that BM chemical toxicity is an important prognostic factor in patients with T4 EC who underwent radical resection after CRT.^25^ The sternum volume exposure to 50 Gy contributed to the BM suppression in breast cancer.^26^ Low‐dose radiation to the pelvis was also significantly associated with hematotoxicity in patients with pelvic tumors, such as cervical cancer and rectal squamous cell carcinoma.^15^, ^27^ However, WU et al. reported that radiation doses to the thoracic vertebrae and ribs in esophageal cancer patients treated with neoadjuvant CRT, including the average dose and the V5–30 of the thoracic vertebrae, and the average dose and V5–20 of the ribs, were related to lymphopenia of grade 3 or above. However, the exposure to the sternum, scapular, and clavicular BM did not affect the occurrence of hematotoxicity.^28^ The reasons for these different results may be due to differences in patient inclusion criteria, treatment mode, statistical methods, and so on.

According to the existing studies, the effect of ionizing radiation on BM is related to the BM microvascular system. Weintraub NL et al. definitively showed a causal relationship between radiation and vascular diseases.^29^ After the BM microvessels are exposed to ionizing radiation, not only is the blood supply of the BM interrupted but also other normal physiological functions may be negatively affected directly or indirectly. In a study by Slayton et al., mice were lethally irradiated with 9.5 Gy and a week after irradiation they lost homeostasis between the extravascular and intravascular space within the BM. The study demonstrates that the marrow sinusoids are damaged by ionizing irradiation, leading to increased sinusoidal diameter and hemorrhage.^30^ A large number of data prove the relationship between low dose radiation (<20 Gy) and acute hematopoietic toxicity. The state of hematopoiesis determines the outcomes of acute radiation syndrome and is critical for the patient's survival.^31^ In our study, we found that the level of ALC2 could predict the outcomes of the patients, we considered that the cumulative dose of radiation at 6–10 times of RT will destroy the homeostasis of BM microvascular, change the state of hematopoiesis and cause acute radiation BM injury in patients, and finally affect the patient's prognosis. However, the above‐mentioned studies are all preclinical studies, and further studies are needed to explain this mechanism.

There are several limitations of this study: (1) all data were derived from a retrospective cohort and we could not standardize the times of blood testing during patient treatment; (2) limited by its sample size, the current data are not generally representative; and (3) there are no standard criteria for delineating BM ranges and different delineation methods may affect the final results. However, this is the first comprehensive retrospective study on the relationships among lymphocyte levels, survival rate, and the sternum and vertebral body BM in patients with unresectable ESCC. Therefore, these results still need to be confirmed in large‐scale prospective studies in the future.

## 5 CONCLUSIONS

In patients with ESCC, the relative volume of sternum BM irradiated by more than 20 Gy was associated with lymphocyte. Patients with ALC2 ≥ 0.8 × 10^9^/L had a significantly higher OS. The V20 of the sternum BM, the V50 of the vertebra body BM, and the level of ALC2 were significant prognostic factors in ESCC patients.

## ETHICS APPROVAL AND CONSENT TO PARTICIPATE

The ethics committee of Shandong Cancer Hospital and Institute approved this study. The study was conducted in agreement with the Declaration of Helsinki. All the patients signed a consent to share their clinical data and information for clinical studies.

## CONFLICT OF INTEREST

The authors have no conflict of interest to report.

## Data Availability

Data are available upon request.
